# Beyond the Human Binary: Decoding Hormone-Immune Plasticity in Transgender Health

**DOI:** 10.3390/biology15141187

**Published:** 2026-07-18

**Authors:** Giuseppa Cembalo, Margherita Turrini, Simone Baldi, Amedeo Amedei

**Affiliations:** Department of Experimental and Clinical Medicine, University of Florence, 50134 Florence, Italysimone.baldi@unifi.it (S.B.)

**Keywords:** gender-affirming hormone therapy (GAHT), immune plasticity, sex steroids, transgender health, microbiome

## Abstract

Sex- and gender-based immune differences have often been interpreted through a male–female biological binary, overlooking how endocrine signaling dynamically shapes immune function. Gender-affirming hormone therapy (GAHT) offers a unique physiological model to disentangle the effects of sex steroids from chromosomal background and examine immune plasticity in contexts relevant to reproductive health. This hormone-informed framework proposes that estradiol and testosterone regulate immune set-points across innate, adaptive, metabolic, and mucosal compartments. Through genomic and non-genomic signaling via androgen and estrogen receptors (AR, ERα/β), these hormones drive distinct immune outcomes: testosterone dampens type I interferon responses in plasmacytoid dendritic cells and reshapes monocyte inflammatory profiles, while estradiol promotes macrophage polarization and enhances T helper1 (Th1) responses. Hormonal effects are closely coupled to cellular metabolism: androgen signaling acts as a “metabolic brake” on Th17 cells by limiting glutaminolysis, a process reinforced by epigenetic remodeling, and is reflected in shifts in the circulating metabolome, positioning metabolomics as a sensitive tool for monitoring hormone-driven immune adaptation. Regardless, hormones also reshape mucosal barriers and reproductive microbiome composition. GAHT alters vaginal and gut microbial communities and their metabolism, influencing mucosal immunity, local inflammation, and reproductive tract homeostasis, with potential implications for fertility preservation, susceptibility to reproductive tract infections, and long-term genital mucosal health. Collectively, this evidence underscores that human immunity is highly responsive to endocrine context. This review synthesizes evidence linking endocrine trajectories, tissue microenvironments, reproductive biology, and social determinants of health, aiming to advance understanding of immune plasticity and contribute to a more inclusive framework of human immune diversity.

## Simple Summary

The immune system does not work the same way in all people since it is deeply influenced by hormones such as estrogen and testosterone. Traditionally, immune differences between individuals have been explained using a simple male–female biological divide, but this view is incomplete. Hormones actively regulate how the immune system responds to infections, inflammation, and the body’s own tissues, and these effects can change when hormone levels shift. People undergoing gender-affirming hormone therapy, a treatment in which transgender individuals receive hormones to align their body with their gender identity, provide a valuable opportunity to study how hormones shape immunity independently of genetic sex. Research shows that testosterone tends to reduce certain inflammatory responses, while estrogen strengthens others. These hormonal effects also influence the communities of microorganisms living in the gut and reproductive tract, which in turn affect local immune defenses and reproductive health. This review brings together current evidence on how hormones, body tissues, the microbiome, and social factors interact to shape immune function. Understanding these connections is important not only for improving the health care of transgender individuals, but also for broadening our knowledge of human immune diversity in general, moving toward a more inclusive and precise approach to medicine.

## 1. Introduction

Marked differences between females and males are consistently observed across infectious diseases, vaccine responses, and autoimmune conditions, affecting both disease incidence and clinical outcomes [1,2,3,4]. These differences are commonly described as sex-related effects. However much of this field continues to rely on simplified binary comparisons between females and males, implicitly treating each category as biologically homogeneous and self-explanatory. This oversimplification is increasingly difficult to justify. Immune responses are shaped by the complex interplay of multiple biological and social determinants, including sex chromosomes, endocrine milieu, lifetime environmental exposures, access to healthcare, stigma, chronic stress, and other structural factors that vary across populations [5,6,7,8,9,10]. Reducing this complexity to biological sex alone risks an overly deterministic interpretation of immune variation and obscures the multifactorial mechanisms that drive individual differences in immune function. A further source of confusion is the frequent use of the terms sex and gender as interchangeable concepts. According to the World Health Organization (WHO), sex refers to the biological characteristics associated with females and males, while acknowledging that these characteristics are not universally binary or mutually exclusive. Gender, in contrast, encompasses the socially constructed roles, behaviors, identities, and expectations that vary across cultures and evolve over time [10]. Distinguishing these concepts is essential for immunological research, as conflating biological and sociocultural determinants may obscure the mechanisms underlying immune variation and overlook the lived experiences of transgender and gender-diverse individuals.

Focusing on transgender health is therefore not a niche add-on to immunology, but both a scientific necessity and an ethical imperative. Transgender individuals experience a disproportionate burden of adverse health outcomes driven by stigma, discrimination, and structural inequities, including reduced access to preventive and chronic psychosocial stress. Each of these factors has the potential to shape immune function through sustained neuroendocrine and inflammatory pathways [8,9].

At the same time, gender-affirming hormone therapy (GAHT) represents a medically necessary intervention for many transgender individuals. Beyond its well-established benefits in reducing gender dysphoria and improving psychological well-being and quality of life, GAHT also provides a unique opportunity to investigate how sustained endocrine changes influence human immune function [11,12]. Research on immunity in transgender populations should therefore be guided by two complementary objectives: first, to elucidate how GAHT and changing endocrine trajectories modulate immune pathways; second, to interpret these biological effects within the broader social and environmental contexts that may amplify or mitigate health risks [8,9,13].

From a human research perspective, GAHT provides a unique physiological model for investigating immune plasticity because it induces sustained changes in circulating sex-steroid concentrations within the same individuals, allowing the effects of hormonal exposure to be examined against a relatively stable chromosomal background [4]. However, conclusions regarding causal mechanisms remain limited, as the current evidence is derived predominantly from longitudinal and observational studies rather than interventional designs [4]. Longitudinal, systems-level studies in transgender men receiving testosterone have revealed coordinated remodeling of innate and antiviral immune programs, characterized by attenuation of type I interferon (IFN-I) responses together with enhanced monocyte inflammatory activity, including increased TNF, IL-6 and IL-15 production, activation of NFκB-regulated pathways, and elevated IFN-γ responses in natural killer (NK) cells [4]. More focused investigations have identified plasmacytoid dendritic cells (pDCs) as particularly sensitive to androgen exposure. Human longitudinal studies consistently demonstrate that testosterone reduces TLR7/8-induced type I interferon production and the expression of downstream interferon-stimulated genes, although the magnitude of these effects varies among individuals [14].

At the vascular–immune interface, testosterone has also been associated with increased leukocyte–endothelium interactions, enhanced expression of adhesion molecules, and elevated inflammatory mediators (IL-6, TNF-α), findings that may have implications for long-term cardiometabolic risk assessment during GAHT [15].

Evidence from transgender women further indicates that immune remodeling is highly tissue-specific and cannot be inferred from peripheral blood alone.

At mucosal sites critical to HIV transmission, neovaginal tissue following penile inversion vaginoplasty exhibits a more inflammatory microenvironment, characterized by increased immune activation, inflammation-associated microbiome profiles, and higher frequencies of CCR5-expressing CD4^+^ target cells. Similar epigenetic signatures suggesting increased CCR5 expression have also been reported in gut mucosa, supporting the concept that localized immune remodeling may influence biological susceptibility to infection [10,16,17]. Crucially, these biological observations must be interpreted alongside behavioral and structural determinants of health, including stigma, healthcare access, and social vulnerability. Rather than acting independently, biological and social factors interact to shape immune responses, disease susceptibility, and clinical outcomes [8,9,10].

The available evidence should also be interpreted in light of its methodological limitations. Most human data derive from relatively small observational or longitudinal cohorts, whereas many mechanistic insights into hormone-dependent immune regulation originate from animal models or in vitro studies. Consequently, direct extrapolation of mechanistic findings to individuals receiving GAHT should be made with caution [13,18].

Moreover, adolescents and young adults remain underrepresented, despite undergoing dynamic endocrine and immune maturation. Only recently have studies begun to characterize immune cell composition in GAHT-treated youth and relate these changes to measured estradiol and testosterone concentrations [19]. These limitations do not decrease the importance of the field; rather, they highlight priorities for future research. Careful characterization of GAHT, including hormone formulation, dose, route of administration, treatment duration, and circulating hormone concentration, should become standard practice. Because endocrine exposure is determined not only by the prescribed regimen but also by inter-individual variability in hormone metabolism and pharmacokinetics, immune outcomes should preferentially be interpreted in relation to measured hormone concentrations rather than treatment categories alone [5,19].

To provide a structured overview of the available evidence, this narrative review critically appraises peer-reviewed studies investigating the effects of GAHT on immune function. The literature considered includes human observational and longitudinal studies together with mechanistic evidence from animal models and in vitro investigations, where these provide biological insight into hormone-dependent immune regulation. Human studies directly evaluating immune outcomes during GAHT are summarized in Table 1 to facilitate interpretation of the current clinical evidence and to distinguish it from the complementary preclinical findings discussed throughout this review. Based on the currently available evidence, this review adopts a hormone-informed framework in which sex steroids are viewed as dynamic regulators of immune function rather than static modifiers of a fixed immune baseline.

Based on the current body of evidence, this review adopts a health-centered, hormone-informed framework in which sex steroids are viewed not as minor modifiers superimposed on a fixed immune baseline, but as dynamic regulators capable of reshaping immune set points across antiviral, inflammatory, and tissue-specific compartments [4,14,15]. Advancing transgender immunology will therefore require an interdisciplinary approach integrating immunology, endocrinology, clinical medicine, and social sciences. Future studies should combine longitudinal immune profiling with rigorous characterization of hormone exposure and careful assessment of behavioral, environmental, and structural determinants of health. Such an integrated framework will not only improve our understanding of immune regulation during GAHT but also provide a stronger evidence base for equitable, personalized, and biologically informed transgender healthcare [4,8,9,10,13].

## 2. Sex Hormone Signaling in Immune Cells: Beyond Classical Endocrinology

Sex hormones, primarily estrogen and testosterone, exert profound immunomodulatory effects that are particularly relevant in transgender individuals receiving GAHT. The endocrine environment established by exogenous hormone administration represents a unique physiological condition capable of reshaping immune cell development, distribution, and function. Nevertheless, despite the recognized role of sex steroids in immune regulation, the immunological consequences of GAHT remain incompletely characterized. Current evidence is still limited, particularly in adolescents and young adults, whose immune and endocrine systems are undergoing dynamic maturation. This knowledge gap is especially important because prolonged hormonal exposure during these developmental stages may have distinct immunological consequences compared with adulthood.

In this regard, the Gender and Immunity (GIM) study represents one of the first efforts to address this knowledge gap by characterizing immune cell composition in young transgender individuals receiving GAHT compared with age-matched controls not undergoing hormone treatment [21]. The study enrolled 100 participants, including 47 transgender individuals, transgender men (assigned female at birth and receiving testosterone) and transgender females (assigned male at birth and receiving estrogen), who had been on GAHT for at least six months, together with 53 control participants. Immune phenotyping was performed using an 18-colour flow cytometry panel, while a subset of samples (*n* = 36) was further characterized using a 37-parameter mass cytometry (CyTOF). Immune cell frequencies were subsequently correlated with circulating estradiol and testosterone concentrations, enabling immune profiles to be interpreted according to endocrine exposure rather than treatment status alone [21]. At the molecular levels, sex steroids regulate immune function not only through systemic endocrine effects but also via direct receptor-mediated signaling within immune cells. These actions are mediated primarily by the androgen receptor (AR) and the classical estrogen receptors (ER) α and β, together with membrane-associated receptor pools that mediate rapid non-classical signaling. Importantly, receptor expression differs substantially across leukocyte subsets, conferring cell-type–specific sensitivity to hormonal stimulation and contributing to the heterogeneous immune responses observed across tissues and inflammatory settings [22,23,24,25].

### 2.1. Androgen Receptor Expression

AR signaling has emerged as a central regulator of innate immune homeostasis. Longitudinal human studies in transgender men receiving testosterone during GAHT have demonstrated coordinated attenuation of IFN-I pathways together with enhanced TNF/NF-κB–associated inflammatory programs, consistent with active AR signaling in circulating immune cells [4,14].

Receptor profiling further indicates that AR expression is highest in pDCs and monocytes, whereas conventional T and B lymphocytes exhibit substantially lower basal AR expression, suggesting a hierarchical pattern of androgen responsiveness across cell lineages [4,14].

### 2.2. Estrogen Receptor Expression

Estrogen receptors are broadly expressed throughout the immune system but show considerable variability in subtype distribution and relative abundance. Both ERα and ERβ are detected in neutrophils, monocytes/macrophages, dendritic cells, and lymphocytes [26,27,28,29,30]. Functional and mechanistic studies, conducted primarily in murine models and in vitro immune cell systems, indicate that estrogen signaling is highly context- and cell-dependent rather than uniformly immunosuppressive or immunostimulatory. For example, estrogen enhances the secretion of MCP-1, MCP-5, eotaxin, and SDF-1 by activated splenocytes, while exerting little or no effect on RANTES, TARC, and KC, underscoring receptor- and gene-specific transcriptional regulation [22,24,31,32,33]. This functional selectivity is likely explained by the heterogeneous distribution of ERα and ERβ across immune cell subsets together with differences in the availability of transcriptional cofactors, resulting in cell type-specific responses to estrogen signaling [24].

**Figure 1:**
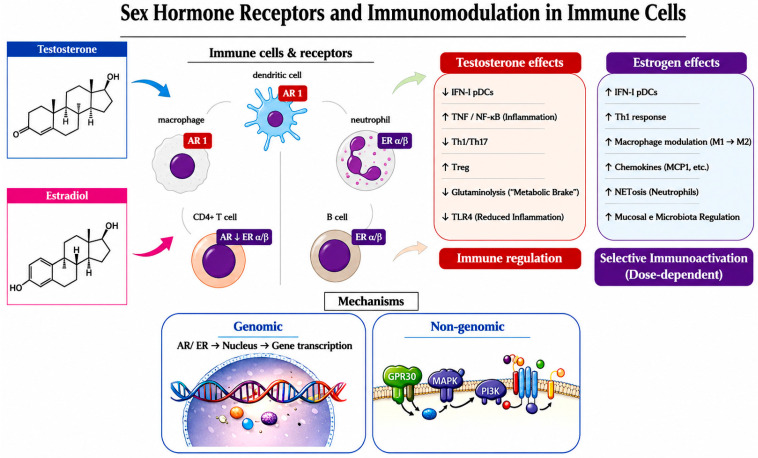
Schematic overview of sex hormone receptor expression and immunomodulatory effects across immune cell subsets. AR and ERα/β are differentially expressed among leukocyte populations, resulting in cell-specific responses to hormonal signaling. Testosterone generally suppresses IFN-I responses, limits Th1 and Th17 differentiation, and promotes Treg expansion, partly through metabolic reprogramming. In contrast, estrogen enhances interferon production, Th1 polarization, macrophage activation, and chemokine secretion in a dose-dependent manner. These effects are mediated through both genomic and non-genomic signaling pathways, underscoring the context-dependent role of sex steroids in immune regulation.

The cell type-specific distribution of sex steroid receptors and their principal immunomodulatory functions are summarized in Figure 1.

## 3. Cell-Type Specificity of Hormone Responsiveness

Although substantial progress has been made in defining hormone-dependent signaling pathways in immune cells, the strength of the available evidence varies across experimental systems. Longitudinal studies in transgender individuals receiving GAHT have documented coordinated immune remodeling, whereas most mechanistic insights derive from experimental animal models and in vitro systems. Consequently, the mechanisms discussed below should be interpreted as biologically plausible frameworks rather than definitive evidence of causal immune changes during GAHT.

### 3.1. T Lymphocytes

Although AR expression is relatively low in T lymphocytes, androgen exposure can substantially reshape T-cell function. In transgender men, testosterone therapy has been associated with increased expression of inhibitory receptors, including TIGIT, together with enrichment of IFN-γ- and TNF-related transcriptional programs, suggesting indirect, or possibly low-level direct, androgenic regulation of T-cell differentiation and exhaustion pathways [4]. Consistent with these findings, immune profiling studies have reported increased proportions of naïve T cells and CD4^+^ regulatory T (Treg) cells following GAHT, with Treg abundance positively correlating with circulating testosterone concentrations, supporting a role for androgens in promoting peripheral immune tolerance [21].

### 3.2. Macrophages

Macrophages represent one of the most hormone-responsive immune cell populations. Evidence from controlled in vitro polarization systems and experimental animal models indicates that estradiol suppresses pro-inflammatory gene expression and IL-1β production while promoting anti-inflammatory markers and enhanced migratory capacity, consistent with an anti-atherogenic phenotype [23,34,35]. Testosterone similarly reduces IL-1β secretion and promotes anti-inflammatory transcriptional programs; however, its effects appear more heterogeneous and highly context-dependent, likely reflecting interactions with macrophage polarization states, tissue microenvironment, and inflammasome activation [23,36,37].

### 3.3. Dendritic Cells and Plasmacytoid Dendritic Cells

pDCs exhibit one of the most pronounced sex differences in innate immunity owing to their robust TLR7-mediated IFN-I production. In trans men, testosterone administration progressively reduces IFN-α/β secretion following TLR7/8 stimulation and attenuates interferon-stimulated gene expression in peripheral blood mononuclear cells [14]. Complementary immune profiling studies have also demonstrated GAHT-associated changes in dendritic cell and monocyte subsets, with several alterations correlating more strongly with circulating testosterone than estradiol levels, even in trans women. These findings suggest that suppression of endogenous testosterone may substantially contribute to immune remodeling during feminizing hormone therapy [21]. Collectively, these observations identify pDCs as a particularly hormone-sensitive immune compartment in which androgen signaling modulates antiviral and inflammatory responses.

Together, these findings support the concept that immune cells function as endocrine-responsive units, with hormone responsiveness determined by receptor expression, intracellular signaling networks, and cellular context rather than by circulating hormone concentrations alone [22,23,25].

## 4. Genomic vs. Non-Genomic Hormone Signaling

Sex hormone signaling in immune cells occurs through two complementary and partially overlapping mechanisms: classical genomic regulation mediated by nuclear receptors and rapid non-genomic signaling initiated at the plasma membrane.

In the canonical genomic pathway, ligand-bound AR or ERα/ERβ translocate to the nucleus, where they regulate the transcription of genes encoding cytokines, chemokines, receptors, and other immunoregulatory proteins. This mechanism underlies estrogen-dependent induction of chemokine networks in lymphoid cells [24], testosterone-mediated repression of B-cell activating factor (BAFF) production and splenic B-cell survival through neuro-stromal circuits [25], and the suppression of inflammatory gene expression and IL-1β production in macrophages, thereby limiting plaque-promoting activity [23].

In parallel, sex hormones also activate rapid signaling pathways through membrane-associated receptor pools, including membrane-localized ER isoforms and the G-protein–coupled estrogen receptor 1 (GPER) [38]. These non-genomic pathways regulate intracellular calcium flux, MAPK and PI3K/Akt signaling, and cellular metabolism independently of de novo gene transcription.

In vitro studies in human neutrophils indicate that estradiol suppresses fMLP-induced migration and superoxide production by inducing the MAPK phosphatase MKP-2 and limiting ERK phosphorylation via classical ERs rather than GPER [26,39,40,41]. Conversely, estrogen promotes NOX-independent neutrophil extracellular trap (NET) formation through GPER-dependent upregulation of peptidyl arginine deiminase 4 (PAD4) and subsequent histone citrullination during neutrophil differentiation [30,42,43].

Membrane-initiated estrogen signalling has also been extensively characterized in non-immune cells. In endometrial carcinoma cells, membrane-impermeable estradiol (E2–BSA) rapidly induces calcium influx and ERK1/2 phosphorylation without activating transcription of survival genes such as BCL2, demonstrating that these rapid signaling events are functionally distinct from classical genomic responses [44]. Similar calcium- and kinase-dependent pathway cascades have been reported in endothelial, vascular smooth muscle, hepatic, intestinal, and tumor cells [45,46].

Collectively, these findings indicate that genomic signaling primarily orchestrates long-term transcriptional programs controlling cytokine production and immune cell differentiation, whereas non-genomic signaling enables rapid regulation of intracellular signaling, metabolism and immediate inflammatory responses. The integration of these complementary pathways provides a mechanistic framework for understanding why sex hormones exert highly context-dependent immunomodulatory effects rather than acting as uniformly immunosuppressive or immunostimulatory mediators. Moreover, the receptor- and cell-specific organization of these signaling networks helps explain the pronounced sexual dimorphism observed across autoimmune, inflammatory, and cardiovascular diseases [22,23,25].

## 5. Endocrine Shaping of CD4^+^ T-Cell Differentiation

A major limitation of the current literature on hormonal immunomodulation in transgender health is the disproportionate emphasis on CD4^+^ rather than CD8^+^ T lymphocytes. This imbalance reflects the current evidence base rather than an intentional exclusion of CD8^+^ immunity.

CD4^+^ T cells have been more extensively investigated because of their central regulatory role in adaptive immunity and their ability to differentiate into functionally distinct subsets, including Th1, Th2, Th17, and Tregs, which provide well-established markers of immune polarization [47,48]. Moreover, much of the immunological research involving transgender individuals (particularly trans women) has originated from HIV-related studies, in which CD4^+^ T cells are the primary viral targets and have therefore been longitudinally monitored in detail [8]. Similarly, the study of hormone-sensitive autoimmune diseases, which are more prevalent in individuals with estrogen-dominant hormonal profiles, has further reinforced the focus on CD4^+^ T cells as key mediators of immune dysregulation [49].

By contrast, the effects of GAHT on CD8^+^ T-cells remain poorly characterized. Although sex hormones are recognized modulators of cellular immunity, current evidence provides only limited phenotypic and functional characterization of CD8^+^ T cells in transgender individuals. Available studies suggest that testosterone therapy in trans men is associated with reductions in CD161^+^ effector memory CD8^+^ T cells [21], whereas estradiol may promote activation profiles resembling those observed in cisgender women [20]. However, major knowledge gaps remain regarding the effects of GAHT on CD8^+^ T-cell senescence, exhaustion, and long-term functionality. In particular, it is still unknown whether hormonal remodeling influences the expression of immune checkpoint molecules such as PD-1, CTLA-4, and LAG-3, or affects cytotoxic activity and memory formation, two processes that are fundamental for antiviral immunity and tumor surveillance [4,47,50]. Consequently, although the following discussion primarily focuses on CD4^+^ T-cell differentiation, the molecular mechanisms described, including metabolic and epigenetic programming, may ultimately provide a conceptual framework for understanding endocrine regulation of CD8^+^ T-cell function as this field evolves. Despite these limitations, accumulating evidence indicates that sex hormones profoundly influence CD4^+^ T-cell differentiation by regulating the balance between inflammatory Th1/Th17 cells and immunosuppressive Tregs, thereby contributing to sex differences in susceptibility to immune-mediated diseases [47,50,51].

Testosterone and AR signaling consistently constrain inflammatory CD4^+^ T cell polarization. Human in vitro studies have shown that testosterone suppresses TNF and IFN-γ production by activated CD4^+^ T cells, while longitudinal studies in transgender men receiving GAHT demonstrate reduced Th1 and Th17 differentiation accompanied by increased Treg frequencies [52].

Mechanistic evidence from mouse models indicates.

These observations are supported by experimental evidence from T cell-specific AR knockout mice, which exhibit enhanced CD4^+^ T-cell proliferation together with preferential differentiation toward Th1 and Th17 phenotypes, resulting in increased production of IL-17A, IL-22, and IFN-γ under polarizing conditions [52].

These findings indicate that androgen signaling acts as an intrinsic regulator limiting inflammatory CD4^+^ T-cell differentiation. Their clinical relevance is further supported by observations in primary biliary cholangitis, where reduced circulating testosterone levels are associated with a pro-inflammatory CD4^+^ T-cell profile characterized by elevated IFN-γ and TNF production [52].

Conversely, estradiol promotes Th1 differentiation under physiological exposure conditions. Continuous low-dose 17β-estradiol (E2) administration in ovariectomized mice significantly increases antigen-specific CD4^+^ T cell expansion while selectively enriching IFN-γ–producing cells without affecting Th2 differentiation cells [53]. This response is maintained across different adjuvant systems and is accompanied by increased expression of Th1-associated markers, including IL-18R [53]. At the clonal level, E2 also expands antigen-specific CD4^+^ T cell populations expressing a public TCR β-chain CDR3 motif, indicating that estradiol preferentially amplifies existing Th1 responses rather than broadening antigen specificity [53].

Collectively, these findings support a model in which testosterone promotes immune tolerance by limiting Th1/Th17 differentiation and favoring Treg development, whereas estradiol enhances Th1 priming and effector expansion under physiological conditions [52,53].

Beyond regulating cytokine production and T-cell subset distribution, sex hormones also imprint CD4^+^ T-cell fate through coordinated metabolic and epigenetic programming. In experimental models of airway inflammation, AR signaling suppresses Th17-mediated pathology by reducing glutamine uptake and glutaminolysis through downregulation of the glutamine transporters ASCT2 (Slc1a5) and SNAT1 (Slc38a1), thereby limiting mitochondrial respiration and IL-17A production [51]. These metabolic changes are accompanied by increased deposition of the repressive histone mark H3K27me3 in regions associated with glutamine metabolism and mitochondrial function, supporting a mechanism in which androgen signaling establishes durable epigenetic programs that restrict inflammatory T-cell differentiation through KDM6-dependent chromatin remodeling [54,55,56]. Together, these findings suggest that androgen signaling promotes long-lasting suppression of inflammatory metabolic circuits while favoring regulatory T-cell differentiation.

The effects of estradiol likewise display marked receptor specificity. The enhancement of Th1 responses requires ERα, but not ERβ, and bone marrow chimera experiments demonstrate that ERα expression in hematopoietic cells is essential for estrogen-mediated expansion of antigen-specific CD4^+^ T cells [53].

Although these studies do not identify individual downstream target genes, they clearly establish classical nuclear estrogen receptors as lineage-defining transcriptional regulators that generate persistent gene expression programs in immune cells following hormonal stimulation [47,53].

Estrogen can also indirectly influence adaptive immunity through myeloid cells. In human peripheral blood mononuclear cell cultures, estradiol enhances spontaneous IgG and IgM production without affecting lymphocyte proliferation or viability. Neutralization of IL-10 partially suppresses this effect, while estradiol preferentially enhances IL-10 production by monocytes rather than T or B lymphocytes, indicating that estrogen-dependent transcriptional reprogramming of myeloid cells contributes indirectly to downstream humoral immune responses [57].

Collectively, current evidence indicates that sex hormones regulate CD4^+^ T-cell differentiation through integrated transcriptional, metabolic, and epigenetic mechanisms rather than through isolated modulation of cytokine production. By coordinating nuclear receptor signaling, metabolic rewiring, chromatin remodeling, and cytokine-mediated myeloid–lymphocyte cross-talk, hormonal cues establish durable, context-dependent biases in Th1, Th17, and Treg differentiation that ultimately shape immune tolerance and inflammatory responses.

## 6. Immunometabolism as a Central Node of Sex-Specific Immunity

### 6.1. Androgen Regulation of T Cell Metabolism

Immunometabolism has emerged as a central mechanism through which sex hormones shape immune cell function and contribute to sex-specific inflammatory outcomes [47,58]. Among CD4^+^ T-cell subsets, Th17 cells provide one of the clearest examples of endocrine regulation of cellular metabolism, with AR signaling limiting glutaminolysis and mitochondrial bioenergetics, thereby restrainingTh17-mediated inflammation [51].

Evidence from human samples, supported by mechanistic studies in experimental mouse models, indicates that male-derived Th17 cells exhibit lower glutaminolytic activity than their female counterparts. Experimental studies further demonstrate that AR signaling suppresses glutamine utilization and mitochondrial respiration, reducing the metabolic capacity required to sustain IL-17A production and inflammatory effector function [51].

Consistent with these observations, models of allergen-induced airway inflammation show that Th17-driven pathology in females depends more heavily on glutamine metabolism than in males, revealing a sex hormone-dependent metabolic vulnerability that becomes apparent following disruption of glutaminolytic pathways [51].

Although these mechanisms have not yet been directly demonstrated in transgender individuals receiving GAHT, they provide a biologically plausible framework through which testosterone therapy could reduce inflammatory potential by reprogramming T-cell metabolism. Reduced glutamine uptake and catabolism limit carbon flux into both the tricarboxylic acid (TCA) cycle and glutathione biosynthesis, impairing mitochondrial bioenergetics and redox buffering while favoring regulatory programs that constrain Th17 differentiation and effector function [59,60]. Together, these findings support the concept that sex steroids regulate immune responses not only through transcriptional control of cytokine expression but also through coordinated modulation of cellular metabolism and oxidative homeostasis during immune activation [58,60].

### 6.2. Metabolic Reprogramming and Immune Plasticity

Hormone-dependent immune plasticity is closely linked to the regulation of nutrient availability and intracellular metabolic pathway utilization. In Th17 cells, AR signaling reduces glutamine uptake through downregulation of the transporters ASCT2 (SLC1A5) and SNAT1 (SLC38A1), thereby limiting substrate availability for glutaminolysis and constraining mitochondrial oxidative metabolism [51]. Because glutamine-derived carbon fuels both the TCA cycle and glutathione-dependent antioxidant defenses, regulation of these transporters represents an upstream metabolic checkpoint controlling inflammatory cell fate.

Metabolomic studies further suggest that inhibition of glutaminolysis promotes compensatory engagement of alternative metabolic pathways, including arginine and serine metabolism, which support polyamine synthesis and one-carbon metabolism [58,59]. These adaptive responses appear to be stabilized through epigenetic remodeling, including increased deposition of the repressive histone mark H3K27me3 at genes involved in mitochondrial function and cellular metabolism [51,55]. Such observations suggest that hormonal regulation extends beyond transient metabolic adaptation, establishing more persistent transcriptional programs that shape immune cell identity and function. Collectively, these findings position immunometabolism as a key point of convergence between endocrine and immune signaling, integrating hormonal cues with metabolic and epigenetic regulation to determine immune cell plasticity [58]. While these mechanisms have been primarily characterized in CD4^+^ T-cell subsets, determining whether comparable metabolic checkpoints regulate the differentiation, persistence, and exhaustion of CD8^+^ cytotoxic T cells during GAHT represents an important priority for future research, particularly given their central roles in antiviral immunity and tumor surveillance [51,61].

## 7. Sex Hormones as Gatekeepers of Innate Immune Cell Plasticity and Function

### 7.1. Hormone-Driven Macrophage Polarization

As discussed above, sex hormones are major regulators of macrophage polarization through activation of androgen and estrogen receptors and their downstream signaling pathways. The effects of estradiol on macrophage function are highly concentration-dependent. At physiological concentrations (approximately 0.1 nM), estradiol enhances TLR4-mediated inflammatory responses through ERα signaling, promoting the expression of IL-6, IL-1β, and inducible nitric oxide synthase (iNOS) while suppressing PI3K/Akt signaling, thereby favoring classical M1 polarization [62].

In contrast, the supraphysiological estradiol concentrations characteristic of pregnancy and the late estrous cycle suppress TNF-α and IL-6 production and promote alternative M2 polarization, supporting tissue repair and the resolution of inflammation.

Beyond regulating inflammatory gene expression, estrogen signaling also remodels macrophage chromatin through DNA methylation and histone modifications, establishing hormone-dependent transcriptional programs that influence long-term macrophage plasticity [62]. Consistent with this mechanism, ERα-dependent alternative activation promotes cutaneous wound healing and coordinated tissue regeneration [63].

Recent studies have further refined this model by demonstrating that estradiol–ERα signaling directly regulates chromatin accessibility at enhancers controlling anti-inflammatory genes [64].

ERα promotes the recruitment of histone acetyltransferases, creating a permissive chromatin landscape that facilitates expression of pro-resolving mediators. These epigenetic changes are closely linked to mitochondrial fitness, as ERα activation preserves oxidative metabolism and prevents the metabolic dysfunction typically associated with chronic inflammatory environments. Collectively, these findings suggest that estradiol establishes sustained transcriptional and metabolic programs that enhance macrophage plasticity and tissue homeostasis rather than simply inducing transient anti-inflammatory responses.

Testosterone exerts complementary regulatory effects that generally limit macrophage inflammatory activation. In addition to suppressing 5-lipoxygenase–dependent leukotriene synthesis, AR signaling downregulates TLR4 surface expression, thereby reducing macrophage responsiveness to lipopolysaccharide (LPS) and other danger-associated molecular patterns. This results in decreased TNF-α production and promotes polarization toward a more anti-inflammatory M2 phenotype while limiting excessive inflammatory tissue damage [65].

Longitudinal studies of GAHT provide evidence that these mechanisms may also operate in humans. Multi-omics analyses have shown that testosterone administration in transgender men progressively recalibrate monocyte-derived macrophage responses, shifting both their transcriptional and epigenetic profiles toward patterns resembling those observed in cisgender males [66].

Although additional longitudinal studies are required, these findings support the concept that the macrophage identity remains highly plastic and continuously adapts to the prevailing endocrine environment through coordinated transcriptional, epigenetic, and metabolic regulation.

### 7.2. Plasmacytoid Dendritic Cells and IFN-I Bias

pDCs are the principal producers of IFN-I during antiviral immune responses through activation of endosomal TLR7 and TLR9 signaling pathways. Among innate immune cells, pDCs exhibit one of the most pronounced examples of sex-specific immune regulation, with estrogen generally enhancing, and androgens suppressing, IFN-I production. ERα plays a central role in this sexually dimorphic response by regulating transcriptional and epigenetic programs controlling interferon production. In addition to directly activating IFNA and IFNB transcription, ligand-bound ERα recruits’ chromatin-remodeling complexes to interferon-associated regulatory regions, increasing histone acetylation and promoting an open chromatin configuration that facilitates rapid transcriptional responses [63,67]. Consistent with this mechanism, recent epigenomic analyses demonstrate that female pDCs display greater chromatin accessibility at TLR7-responsive enhancers together with increased ERα occupancy at IRF5 and IRF7-regulatory loci [68].

The dynamic nature of this regulatory network is illustrated by longitudinal studies of GAHT. Testosterone administration in transgender men progressively reduces IFN-α production following TLR7/8 stimulation, indicating that hormonal exposure can reshape antiviral immune responses over time [14]. Complementary multi-omics analyses further demonstrate that testosterone therapy induces coordinated transcriptional and epigenetic remodeling within innate immune cells, gradually shifting chromatin organization toward profiles characteristic of cisgender males [4]. Conversely, AR signaling suppresses antiviral responses by recruiting transcriptional corepressors to TLR7- and IRF7-associated regulatory regions, thereby limiting activation of NF-κB and IRF7 signaling pathways [4,69]. The resulting reduction in IFN-I production has been associated with higher viral burdens and poorer clinical outcomes in infections such as influenza and COVID-19 [70,71].

From an evolutionary perspective, this sexually dimorphic regulation represents a classic biological trade-off. Enhanced IFN-I responsiveness in females and in individuals receiving feminizing GAHT may improve antiviral immunity but simultaneously increase susceptibility to interferon-mediated autoimmune diseases, including systemic lupus erythematosus [72,73]. Overall, the balance between ERα-dependent enhancement and AR-mediated repression of interferon signaling provides a mechanistic framework for understanding how sex hormones regulate antiviral defense while influencing autoimmune risk.

## 8. Mucosal Immunity Under Hormonal Control

### 8.1. Genital and Gut Mucosa as Hormone-Sensitive Immune Niches

The gastrointestinal and genitourinary tracts constitute highly specialized immune environments in which epithelial barrier integrity, innate sensing, and adaptive immune responses are continuously shaped by endocrine signals. Growing evidence identifies sex steroids as key regulators of these hormone-sensitive environments, exerting direct influence on mucosal susceptibility to viral pathogens, such as HIV-1, and modulating the trajectory of chronic inflammatory states. However, the relative contribution of direct hormonal effects versus secondary changes in the microbiome or local inflammatory milieu remains incompletely understood.

One of the best-characterized mechanisms involves the regulation of activated CD4^+^CCR5^+^ T cells, the principal target cells for HIV-1 infection. Experimental and observational evidence suggests that estrogens generally reduce mucosal susceptibility by decreasing CCR5 expression and limiting the production of inflammatory chemokines, including CCL2 and CCL5. Consistent with these observations, transcriptomic analyses have associated higher estradiol concentrations with reduced CCR5 availability and a less inflammatory mucosal profile. Conversely, physiological fluctuations in progesterone or exposure to exogenous progestins may transiently increase the abundance of activated CCR5^+^ T cells and plasmacytoid dendritic cells within the cervicovaginal mucosa, potentially creating periods of increased susceptibility to viral acquisition. These observations suggest that reproductive hormone fluctuations dynamically influence mucosal immune homeostasis, although the magnitude of these effects likely varies across individuals and clinical settings [74,75,76,77].

Beyond leukocyte composition, sex steroids also influence epithelial barrier function. Experimental studies indicate that estrogens regulate the expression of tight junction proteins, including claudin-1 and occludin, thereby reducing paracellular permeability and contributing to barrier integrity [78]. Whether comparable mechanisms operate during GAHT remains incompletely established. Preliminary evidence suggests that feminizing GAHT is associated with alterations in gut microbial composition and reduced systemic inflammatory markers, changes that may indirectly support mucosal barrier function [79,80]. Conversely, testosterone therapy has been associated with increased frequencies of circulating regulatory T cells and reduced Th1/Th17 polarization. Nevertheless, emerging clinical observations indicate that androgen exposure may exert tissue-specific effects within the gastrointestinal tract, and some studies have suggested an increased frequency of inflammatory bowel disease flares in susceptible individuals receiving masculinizing GAHT, although causality remains uncertain and larger prospective studies are required [52,81].

Sex steroids further modulate mucosal immunity through regulation of pattern-recognition receptor (PRR) signaling. Estrogens influence the expression and activity of Toll-like receptors (TLRs), particularly TLR3 and TLR7/8, enhancing interferon regulatory factor activation and type I interferon production in epithelial cells and plasmacytoid dendritic cells [82,83,84].

While these responses strengthen antiviral immunity, prolonged or supraphysiological estrogen exposure has been proposed to facilitate excessive inflammasome activation, including NLRP3-dependent pathways, potentially amplifying chronic mucosal inflammation under specific pathological conditions [85,86]. In contrast, androgens generally attenuate PRR-mediated inflammatory responses by inducing negative regulators of TLR signaling, including suppressor of cytokine signaling 1 (SOCS1) and dual-specificity phosphatase 1 (DUSP1), thereby reducing NF-κB activation and downstream production of TNF-α and IL-6 following LPS stimulation [52,87].

Although this anti-inflammatory activity may promote tolerance toward commensal microorganisms, excessive suppression of innate responses could theoretically impair epithelial repair during chronic inflammatory disorders such as inflammatory bowel disease [88]. Progesterone has likewise been implicated in limiting excessive inflammasome activation through modulation of NOD-like receptor signaling, contributing to maintenance of immune tolerance during pregnancy [89,90,91]. Collectively, these findings indicate that sex hormones regulate mucosal immunity through coordinated effects on epithelial barrier integrity, innate immune sensing, and adaptive immune responses in a tissue-specific and context-dependent manner.

### 8.2. The Endocrine–Microbiome–Immune Axis

The structural and cellular organization of hormone-sensitive mucosal niches is inextricably linked to the bidirectional dialogue between sex steroids and the commensal microbiome, a phenomenon termed the “microgenderome.” The estrobolome orchestrates this interplay, functioning as a metabolic rheostat where bacterial β-glucuronidases deconjugate estrogen metabolites to calibrate systemic steroid bioavailability [92]. This reciprocal interaction suggests that endocrine and microbial homeostasis are dynamically interconnected rather than acting as independent regulatory systems.

Longitudinal microbiome studies indicate that microbial composition varies across physiological hormonal states. In cisgender women, fluctuations during the menstrual cycle have been associated with changes in microbial diversity and taxonomic abundance, including increased representation of *Lactobacillus* spp. and several butyrate-producing Firmicutes during estrogen-dominant phases [93,94]. Experimental evidence further suggests that *Faecalibacterium prausnitzii*, one of the principal butyrate-producing commensals, may respond to estrogenic signaling by increasing butyrate production, although the underlying molecular mechanisms remain under investigation [95]. Emerging evidence also indicates that GAHT is accompanied by measurable alterations in gut microbial composition. Preliminary longitudinal studies suggest that feminizing GAHT is associated with increased microbial diversity and enrichment of taxa linked to short-chain fatty acid production, including *Akkermansia muciniphila*, a species implicated in maintenance of epithelial barrier integrity and metabolic homeostasis [96,97,98]. Conversely, masculinizing GAHT has been associated with shifts in microbial communities that include increased abundance of *Ruminococcus gnavus* and reduced representation of *A. muciniphila* [95]. Although these observations suggest that endocrine manipulation contributes to remodeling the intestinal microbiome, current evidence remains limited by relatively small cohorts, heterogeneous treatment regimens, and potential confounding by dietary and lifestyle factors.

**Figure 2:**
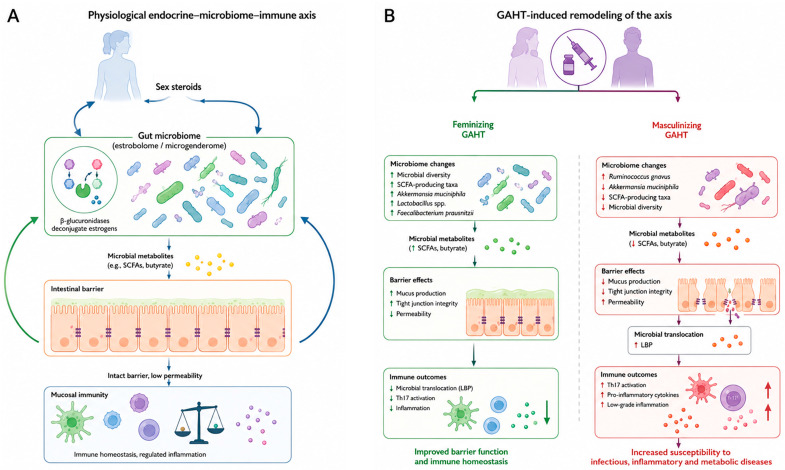
The endocrine–microbiome–immune axis and its remodeling during GAHT. (**A**) Physiological bidirectional interactions between sex steroids, the gut microbiota, intestinal barrier function, and mucosal immunity. The estrobolome regulates estrogen metabolism, while microbial metabolites, particularly short-chain fatty acids (SCFAs) such as butyrate, support epithelial barrier integrity and immune homeostasis. (**B**) Proposed remodeling of this regulatory network during GAHT. Current evidence suggests that feminizing GAHT is associated with enrichment of SCFA-producing bacteria and improved barrier homeostasis, whereas masculinizing GAHT has been associated with reduced abundance of barrier-supporting taxa, increased intestinal permeability, microbial translocation, and enhanced pro-inflammatory immune responses.

Alterations in microbial composition may, in turn, influence mucosal immunity. Reduced abundance of barrier-supporting bacteria has been associated with increased intestinal permeability and elevated circulating lipopolysaccharide-binding protein (LBP), a surrogate marker of microbial translocation [82,99,100,101,102]. Enhanced exposure to microbial products may subsequently promote Th17 differentiation and chronic low-grade inflammation. Experimental models further suggest that sex hormones may preserve intestinal architecture and microbial metabolite production during metabolic or circadian stress [103,104], although confirmation in human longitudinal studies is still required. Overall, current evidence supports the concept that mucosal immunity emerges from continuous interactions among endocrine signals, microbial communities, epithelial barrier function, and immune-cell responses. Rather than acting independently, these components form an integrated regulatory network whose disruption may contribute to susceptibility to infectious, inflammatory, and metabolic diseases (Figure 2). Defining how GAHT modifies each component of this network represents an important priority for future longitudinal human studies.

## 9. Outstanding Questions

### 9.1. Limitations of the Current Evidence

Despite the growing body of literature investigating the immunological effects of GAHT, several important limitations should be acknowledged. First, substantial heterogeneity exists among published studies with respect to hormone formulation, dosage, route of administration, treatment duration, and the concomitant use of androgen-suppressive therapies. These differences complicate direct comparisons across cohorts and limit the generalizability of current findings. Moreover, treatment category alone does not accurately reflect endocrine exposure, as circulating estradiol and testosterone concentrations vary considerably among individuals because of differences in pharmacokinetics, hormone metabolism, treatment adherence, and individual physiology. Future studies should therefore report GAHT regimens in detail and incorporate measured hormone concentrations as continuous variables rather than relying solely on categorical treatment groups.

A second limitation is the relatively small number of prospective longitudinal studies evaluating immune changes before and after GAHT initiation. Although recent high-dimensional immune profiling studies provide compelling evidence that GAHT remodels innate, adaptive, and mucosal immune compartments, most available human studies remain observational and involve relatively small and heterogeneous cohorts. Consequently, causal relationships cannot yet be firmly established, and the immunological changes observed during GAHT should be interpreted as associations requiring further validation.

Finally, direct mechanistic evidence linking GAHT to specific immune outcomes in humans remains limited. Many of the pathways discussed throughout this review, including T-cell metabolic reprogramming, macrophage polarization, type I interferon regulation, mucosal immunity, and microbiome–immune interactions, have been elucidated or in vitro systems. Although these studies provide biologically plausible mechanisms, their relevance to transgender individuals receiving GAHT must be confirmed through prospective human studies integrating standardized hormone monitoring with longitudinal immune profiling, metabolomics, epigenomics, microbiome characterization, and clinically meaningful outcomes.

### 9.2. Outstanding Research Questions

A major unresolved question concerns the persistence of hormone-induced immune remodeling. It remains unclear whether the immunological and microbial changes observed during puberty or following GAHT are fully reversible or instead generate durable epigenetic programs that persist after endocrine conditions change. Emerging evidence suggests that prolonged exposure to sex steroids may induce stable epigenetic modifications, including H3K4me3 enrichment in hematopoietic stem cells, potentially influencing the functional properties of their progeny long after hormonal exposure has ceased [105,106,107]. Consistent with this hypothesis, experimental studies indicate that although immune cell phenotypes may return to baseline following hormone withdrawal, transcriptional priming can persist, suggesting a form of hormone-induced innate immune memory [108,109].

Another important challenge is the need to disentangle the biological effects of sex from the influence of gender-related social and environmental factors.

Future studies should explicitly distinguish biological variables—including chromosomal complement, gonadal function, and circulating hormone concentrations—from gender-associated determinants such as psychosocial stress, healthcare access, lifestyle, and environmental exposures, all of which independently influence immune function. Treating sex and gender as interchangeable variables risks obscuring the mechanisms responsible for observed immune differences and may contribute to inconsistent or misleading conclusions.

One example is chronic psychosocial stress, which activates the hypothalamic–pituitary–adrenal (HPA) axis and increases systemic glucocorticoid concentrations. Through NF-κB transrepression and other immunoregulatory pathways, glucocorticoids profoundly influence leukocyte function and may either amplify or mask the immunological effects of sex steroids [110]. Accounting for these interacting biological and environmental variables will be essential for accurately interpreting immune phenotypes across diverse populations, including individuals whose endocrine profiles do not conform to traditional binary classifications.

Ultimately, advancing toward precision immunology will require integrative, high-dimensional approaches that treat endocrine exposure as a continuous biological variable while simultaneously incorporating gender-related environmental determinants [111]. Additional priorities include identifying reliable biomarkers of hormonal exposure, determining how different GAHT regimens influence immune remodeling, and establishing which immunological changes are transient, adaptive, or persist during long-term hormone therapy.

## 10. Conclusions and Future Perspectives

Current evidence supports the concept that sex steroids are major regulators of immune plasticity, influencing innate, adaptive, metabolic, and mucosal immune compartments through integrated endocrine signaling networks. Human studies, particularly recent longitudinal investigations in individuals receiving GAHT, demonstrate that changes in the hormonal milieu are accompanied by coordinated remodeling of immune-cell composition, interferon responses, inflammatory pathways, and tissue-specific immune phenotypes. Together with complementary mechanistic evidence, these findings support the view that immune function is dynamically shaped by endocrine signals rather than being determined solely by chromosomal background. At the same time, several concepts discussed throughout this review should be considered emerging rather than definitive. Increasing evidence suggests that sex hormones regulate immune metabolism, epigenetic remodeling, and host–microbiome interactions, thereby contributing to long-term immune adaptation. However, much of the mechanistic understanding of these processes still derives from experimental animal models and in vitro systems, whereas direct validation in longitudinal cohorts of individuals receiving GAHT remains limited. Several key questions therefore remain unresolved. It is still unclear whether hormone-induced immune remodeling generates durable epigenetic memory, whether metabolomic and microbiome signatures can serve as reliable biomarkers of endocrine-driven immune adaptation, and how these molecular changes ultimately influence susceptibility to infection, autoimmunity, cancer, and cardiovascular disease. Likewise, the extent to which immune remodeling varies according to GAHT formulation, hormone concentrations, treatment duration, and age at therapy initiation has yet to be systematically established. Addressing these questions will require well-designed prospective longitudinal studies integrating standardized documentation of GAHT regimens, serial hormone measurements, high-dimensional immune phenotyping, multi-omics technologies, and clinically relevant outcomes. Such integrative approaches will be essential to distinguish causal mechanisms from associative observations and to translate experimental findings into evidence-based clinical practice. Overall, the available evidence supports a paradigm in which the immune system is best understood as a highly plastic, endocrine-responsive network that continuously adapts to the prevailing hormonal environment. Viewing immunity through this hormone-informed framework not only advances our understanding of transgender health but also provides broader insights into the biological basis of sex-related immune diversity. Ultimately, integrating endocrinology, immunology, metabolism, and systems biology may pave the way toward more precise and personalized approaches for the prevention and treatment of immune-mediated diseases across diverse human populations.

## Figures and Tables

**Table 1:** Human studies investigating immune modulation associated with GAHT.

Reference	Population	GAHT	Methodological Approach	Key Findings
pDC function studies [12]	Transgender adults	Testosterone	Functional analysis of plasmacytoid dendritic cells	Reduced IFN-I production, associated with attenuated antiviral responses
IFN studies [13,14]	Transgender adults	Testosterone	Interferon production assays	Decreased IFN production, indicating modulation of innate immune responses
Treg study [15]	Transgender adults	Testosterone	T cell immunophenotyping	Increased Treg frequency, consistent with enhanced immunoregulatory activity
ANA study [16]	Transgender women compared with cisgender individuals and transgender men	Estrogen	Serological analysis	Elevated antinuclear antibody levels in transgender women
SLE case studies [17,18]	Transgender women	Long-term estrogen	Clinical case reports	Reported association with development of systemic lupus erythematosus
MS retrospective study [19]	Transgender women	Estrogen	Retrospective hospital record analysis	Increased risk of multiple sclerosis compared with birth-assigned males
Early youth studies [20]	Adolescents and young transgender individuals	GAHT (estradiol and testosterone)	Immune profiling with hormone correlation	Early evidence linking circulating hormone levels to immune cell variation
Gender and Immunity (GIM) study [21]	100 participants (47 transgender, 53 controls)	Testosterone (transgender men)/Estrogen (transgender women)	18-colour flow cytometry and 37-parameter CyTOF	Distinct immune cell profiles correlated with circulating estradiol and testosterone levels

## Data Availability

No new data were created or analyzed in this study. Data sharing is not applicable to this article.
